# Patients’ experiences and effects of non-pharmacological treatment for myalgic encephalomyelitis/chronic fatigue syndrome – a scoping mixed methods review

**DOI:** 10.1080/17482631.2020.1764830

**Published:** 2020-05-20

**Authors:** Anne Marit Mengshoel, Ingrid Bergliot Helland, Mira Meeus, Jesus Castro-Marrero, Derek Pheby, Elin Bolle Strand

**Affiliations:** a Department of Interdisciplinary Health Sciences, Institute of Health and Society, Faculty of Medicine, University of Oslo, Oslo, Norway; b Norwegian National Advisory Unit on CFS/ME, Division of Pediatrics and Adolescents, Rikshospitalet, Oslo University Hospital, Oslo, Norway; c Research Group MOVANT, Department of Rehabilitation Sciences and Physiotherapy, University of Antwerp, Wilrijk, Belgium; d Department of Rehabilitation Sciences and Physiotherapy, Ghent University, Ghent, Belgium; e Pain in Motion, International Research Group; f CFS/ME Unit, Vall d’Hebron University Hospital Research Institute, Universitat Autònoma De Barcelona, Barcelona, Spain; g Society and Health, Buckinghamshire New University, High Wycombe, UK; h Faculty of Health Studies, VID Specialized University, Oslo, Norway

**Keywords:** Myalgic encephalomyelitis, chronic fatigue syndrome, systematic literature review, cognitive behavioural therapy, activity pacing, exercise, rehabilitation, effect, patient experiences, non-pharmacological treatments

## Abstract

**Purpose:**

The EU COST Action 15111 collaboration on myalgic encephalomyelitis/chronic fatigue syndrome (ME/CFS) aims to assess current research and identify knowledge gaps in Europe. Presently, our purpose is to map the effects of non-pharmacological therapies (NPTs) for ME/CFS, and what patients find important in the treatment process.

**Methods:**

A scoping mixed methods literature review of European studies identified 16 papers fulfiling our inclusion criteria. The quantitative and qualitative studies were synthesized separately in tables. Additionally, extracts from the qualitative studies were subjected to translational analysis.

**Results:**

Effect studies addressed cognitive behavioural therapy (CBT, n = 4), multimodal rehabilitation (n = 2) and activity-pacing (n = 2). CBT reduced fatigue scores more than usual care or waiting list controls. The effects of rehabilitation and activity-pacing were inconsistent. The contents, assessment methods and effects of rehabilitation and activity pacing studies varied. For patients, health professionals’ recognition of ME/CFS and support were crucial, but they expressed ambiguous experiences of what the NPTs entail.

**Conclusions:**

Methodological differences make comparisons across NPTs impossible, and from a patient perspective the relevance of the specific contents of NPTs are unclear. Future well-designed studies should focus on developing NPTs tailored to patients’ concerns and evaluation tools reflecting what is essential for patients.

## Introduction

The EUROMENE collaboration (COST Action 15111) on myalgic encephalomyelitis/chronic fatigue syndrome (ME/CFS) aims to assess current knowledge, identify knowledge gaps, and deliver information on the disease burden, potential biomarkers, clinical diagnosis and potential treatment in Europe (http://www.euromene.eu/). On behalf of EUROMENE, we conducted a scoping mixed methods review on the evidence of effectiveness (quantitative studies) and patients’ experiences of treatment processes (qualitative studies) of non-pharmacological therapies (NPTs) in Europe to identify knowledge gaps for future research in ME/CFS.

ME/CFS is a contested diagnosis because it cannot be explained by any definite pathology, and there is no known cure (Sharpe, 2011). Patients feel ill, but objective clinical tests cannot verify or explain this. The diagnosis is based on reported symptoms and exclusion of other diseases and fatiguing conditions (Fukuda et al., 1994), and diagnostic activities may take time (Hannon et al., 2012). The main symptom of ME/CFS is excessive new onset fatigue and post-exertional malaise (Institute of Medicine, 2015), frequently accompanied by unrefreshing sleep, cognitive dysfunction, and muscle and joint pain (Larun & Malterud, 2007; Strand et al., 2019a). ME/CFS seriously restricts daily living and social participation (Njølstad et al., 2019) which creates burdens for patients and their families (Nacul et al., 2011). Given the lack of curative pharmacological therapy, many patients are frequently referred to NPTs that, in various ways, aim to help patients living with ME/CFS. Studies of NPTs include cognitive behaviour therapy (CBT), which is based on principles of classical and operant learning theories (Davidson, 2008), and aims to modify dysfunctional beliefs and improve patients’ coping skills and behaviour (Price et al., 2008). Another approach, activity pacing, is based on the envelope theory that claims, if expended energy levels are kept relatively constant, patients will slowly recover (Jason, 2008). Thus, the aims are to reduce avoidance behaviour, overexertion and symptom fluctuations through activity-rest cycling, energy conservation, and graded activity (Antcliff et al., 2016). Also offered is graded exercise therapy (GET) that presupposes that exercise avoidance perpetuates symptoms through deconditioning (Clark & White, 2005), and therefore aims to increase the activity level and improve functional capacity via a low-level exercise programme, gradually increasing in intensity. Rehabilitation programmes can be provided, including a mixture of, for example, CBT, GET, activity pacing, mindfulness, relaxation strategies, body awareness therapy, sleep hygiene, stress management, and gradual physical and social reactivation (Thomas et al., 2008).

Over the last decades, systematic literature reviews have reported some effects of NPTs, but the inclusion of patients is based on various diagnostic criteria (Castro-Marrero et al., 2017; Chambers et al., 2006; Edmonds et al., 2004; Kim et al., 2020; Larun et al., 2015; Nijs et al., 2011; Price et al., 2008; Van Cauwenbergh et al., 2012). Studies have not necessarily excluded patients with mental illnesses, for which the results have been widely criticized. As CBT and GET are used for patients with mental illnesses, for example, those with severe depression, it was important for us to find out whether such therapies are found efficient when patients suffering from mental illnesses were explicitly excluded. Therefore, we have reviewed papers based on case definitions in line with the EUROMENE recommendations (Strand et al., 2019b); which explicitly exclude mental illnesses. We also sought to record what were defined as primary outcome variables and which instruments were used for assessments. Furthermore, with a view that effects of NPTs relate to what patients’ find meaningful and do during a treatment process, we also included studies referring interviews with patients about their views about participating in NPTs. Thus, our review aimed to map the effects of NPTs in patients with ME/CFS and to examine what patients find important in the treatment process.

### Recovery as treatment effects and personal healing work

Recovery is an umbrella term with various interpretations related to different understandings of health (Jacobson, 2004). For example, recovery can be seen as an outcome or evidence of a particular therapy’s effect of improving a disease, or alternately, as a personal engagement in a process of coming to terms with or overcoming an illness condition. With respect to NPTs, the patient’s efforts and engagement during a treatment process is essential to arrive at successful outcome of treatment. A dominant understanding of health is absence or reduction of disease that can be assessed in terms of fewer disease-related biological abnormalities and symptoms (Cassell, 2004). Accordingly, recovery is based on a causal logic where an effective treatment should lead to outcomes of reduced disease. Whereas disease relates to objectivity, illness is communicating the subjective, personal experiences of patients of how a disease afflicts and disrupts a self and life (Kleinman, 1988). Thus, recovery can be understood as a personal process of acting upon own illness experiences to bring more wellness into life (Cassell, 2004). This process claims a person’s resources, efforts and engagement (Mattingly, 1998). In different ways, though, the purpose of NPTs is to facilitate such a process. The progress becomes “visible” through people’s story-telling about small and big events (turning points) that make a difference and enable a patient to proceed (Frank, 1995; Mattingly, 1998). Presently, this interpretation informed our analysis of qualitative data.

## Methods

### Design

Three researchers (AMM, EBS, and IBH) planned the present study. A scoping mixed methods review design was considered appropriate for mapping research evidence and identifying knowledge gaps (Munn et al., 2018). The literature search and inclusion/exclusion process followed the procedures of a systematic literature search, but according to a scoping review design, the included papers were not subjected to any methodological quality appraisal (Munn et al., 2018).

### Information sources and search

With the help of a research librarian, a systematic literature search was conducted on the following e-data bases: Medline, Cinahl, PsychInfo, Embase, Cochrane Database, Cochrane Library, Web of Science and Epistemonikos. Separate search strategies for quantitative and qualitative studies were created for Ovid Medline (Table I) and adapted to the other databases. We decided to examine the research trend in Europe over a period of the last ﻿10 years (from January 2009 to January 2019). The reason to include only the last ﻿10 years was that we wanted to examine if there were any new trends in focus for research compared with what described in prior systematic literature reviews. Later, an updated search following prior procedures was performed up till 18 March 2020 without identifying any additional studies.

**Table I.** The search profile applied in Medline being adapted for the other databases.

**Outcome studies**: 1 (Fatigue Syndrome, Chronic/or ((CFS adj3 (ME or SEID)) or myalgic encephalo* or chronic fatigue* or fatigue syndrom* or systemic exertion intolerance or PVFS or royal free disease* or akureyri disease or Iceland disease).tw,kf.) and ((randomis* or randomiz* or randomly or trial or intervention? or effect? or impact? or multicenter or multi centre or multicentre or multi centre or controlled or control group? or (before adj5 after) or (pre adj5 post) or ((pre-test or pre test) and (post-test or post test)) or quasiexperiment* or quasi experiment* or evaluat* or time series or time point? or repeated measur*).ti,ab. or ((treatment outcome/and follow up study/) or ((followup or follow up or longitud* or cohort*) and (treatment* or therap* or intervention* or management)).tw,kf. or (treatment* or therap* or intervention* or management).ti.)) 2 Fatigue Syndrome, Chronic/or ((CFS adj3 (ME or SEID)) or myalgic encephalo* or chronic fatigue* or fatigue syndrom* or systemic exertion intolerance or PVFS or royal free disease* or akureyri disease or Iceland disease).tw,kf. 3 limit 2 to (clinical study or clinical trial, all or clinical trial, phase i or clinical trial, phase ii or clinical trial, phase iii or clinical trial, phase iv or clinical trial or controlled clinical trial or randomized controlled trial) 4 limit 2 to “therapy (best balance of sensitivity and specificity)” 5 1 or 3 or 4 6 limit 5 to (english language and yr = “2009 -Current”) **Qualitative studies about patients’ experiences**: (Fatigue Syndrome, Chronic/or ((CFS adj3 (ME or SEID)) or myalgic encephalo* or chronic fatigue* or fatigue syndrom* or systemic exertion intolerance or PVFS or royal free disease* or akureyri disease or Iceland disease).tw,kf.) and ((((“semi-structured” or semistructured or unstructured or informal or “in-depth” or indepth or “face-to-face” or structured or guide) adj3 (interview* or discussion* or questionnaire*)) or (focus group* or qualitative or ethnograph* or ethnological or fieldwork or field work or field stud* or key informant or focus group* or narrat* or phenomenolog* or hermeneutical or ethonurs* or grounded theory)).tw,kf. or interviews as topic/or focus groups/or narration/or qualitative research/) Limited to English language and from January 2009 up till March 2020

### Eligibility criteria

We included studies of patients ≥12 years old. For quantitative studies addressing our first aim, ME/CFS was determined by the Canadian Consensus Criteria (CCC) (Carruthers et al., 2003), the International Consensus Criteria (ICC) (Carruthers et al., 2011), or the Centre for Disease Control and Prevention (CDC-94) criteria definitions (Fukuda et al., 1994), consistent with EUROMENE recommendations (Strand et al., 2019b). The qualitative studies for the second aim, however, often failed to report diagnostic criteria, so they were included irrespective of case definitions. For the first study aim, only randomized controlled trials (RCTs), and for the second aim, individual or focus group interviews, and in both cases only European patients, were included, to describe the European landscape. Excluded were studies without full text, case reports, pilot studies, protocols for effect studies, non-English language papers, secondary analysis of RCTs, other designs than RCTs including experimental studies of exercise or mental mechanisms, duplicates and non-European studies. In planning the study, we decided to exclude conference reports, proceedings, Master’s and PhD theses as such literature can be difficult to search and retrieve (Peters et al., 2015).

### Study selection

The search strategy identified titles and abstracts. Two authors (EBS, AMM) independently screened the papers for potential eligibility by reading titles and abstracts. Each reference for which initial agreement was not reached were discussed before a final set of references were retrieved and reviewed by the researchers in full-text. After the independent reading, the papers were discussed, and more papers were excluded.

### Research team

The authors have various backgrounds from physiotherapy, psychology, medicine, biology and economy, and we are representing a European collaborative group of researchers from about 20 countries.

### Synthesis and analysis of qualitative data

The synthesis of the studies includes case definition, recruitment methods, type of NPT, primary outcome measures (quantitative methods), data collection, outcomes of primary outcome variable and effect size whenever reported in the papers (quantitative studies) and analytical themes developed by the authors of primary papers (qualitative studies), see Table II for quantitative and Table III for qualitative studies. Additionally, text from the qualitative papers’ result sections addressing patients’ experiences of NPT was identified and extracted. All text, including what patients experienced as unimportant or meaningful by participating in NPTs, was extracted and imported into the software programme NVivo for further analysis. The extracts of each paper were coded and compared across papers for similarities or dissimilarities, in accordance with reciprocal and refutational translational analysis (Britten et al., 2002; Noblit & Hare, 1988). During this process, new categories were developed to capture what was described in the papers as a whole. As our analysis emerged, three overarching themes were developed.

**Table II.** An overview of included papers referring effects of non-pharmacological programmes.

	Aim/research question	Setting for intervention, Diagnostic criteria	Interventions and control	Length of follow-up	Sample characteristics	Primary outcome measures	Statistically significant group differences in primary outcome variable, and effect size if reported
**Cognitive behavioural therapy (CBT) for adults:**							
Janse et al., 2018 Netherlands	To test the efficacy of internet-based CBT (iCBT)	Outpatient specialized clinic CDC-94 criteria †	Protocol-driven- feedback iCBT vs. feedback-on-demand iCBT vs. waiting list	At end of therapy at 6 months	80 patients in each three groups, 145 women. Mean age in groups from 37 to 40 yrs	Checklist Individual Strength fatigue severity subscale	No difference between the two iCBT formats, but both groups better fatigue than controls. 43% in iCBT groups reached normal fatigue and 15% in waiting list control group No serious adverse events
Tummers et al., 2012 Netherlands	To test whether a minimal intervention was effective when delivered in a community-based mental health centre	Community-based mental health centre CDC-94 criteria †	Self-instruction booklet based on CBT and email-contacts once every 2 weeks vs. waiting list	At time of completing intervention and waiting list after 6 months	Guided CBT, n=62, 46 women, mean age 36 yrs, illness duration 6 to 464 months Waiting list, n=61, 50 women, mean age 36 years, illness duration 6 to 625 months	Checklist Individual Strength fatigue severity subscale	Decreased fatigue was found in guided CBT group compared with waiting list controls. Controlled effect size was 0.70. 33% showed clinical reduced fatigue compared to 9% in waiting list control group
Tummers et al., 2010 Netherlands	To examine effectiveness of a stepped care guided self-instruction and CBT compared to usual care	Expert centre of ME/CFS CDC-94 criteria †	Stepped care: Minimal self-instruction booklet followed by 6 months individual face to face CBT vs. waiting list followed by CBT	At completion self-instruction/waiting period. After completing additional CBT or regular CBT	Self-instruction and CBT, n=84, 69 women, mean age 37 yrs Waiting list and regular CBT, n=85, characteristics not reported	Checklist Individual Strength fatigue severity subscale	No statistically significant difference between two groups in fatigue, effect size 1.37 Number of patients reaching clinical significant improvement higher in the stepped care group than the other group
**CBT for adolescents:**							
Nijhof et al., 2012 Netherlands	To compare short-term effectiveness of the FITNET programme with usual care in reduction of fatigue, school absence, and physical dysfunction	Outpatient specialist care CDC-94 criteria †	CBT delivered as 21 modules over internet, with interactions between trained CBT therapists and children and their parents separately first once a week and then each other week vs. usual care controls	At 6 months	CBT group, n=68, usual care n= 67, CBT group, 54 girls, mean age 16 yrs and duration of symptoms 16 yrs Usual care group, 57 girls, mean age 16 yrs and duration of disease 19 yrs.	School attendance in % of normal	School attendance in CBT group increased to 84% vs 51% in usual care group, and 75% reached full school attendance at 6 months vs 16% in usual care group.
**Rehabilita-tion:**							
Nunez et al., 2011 Spain	To compare quality of life of those receiving conventional drugs, group CBT and GET with drug and exercise counselling	Outpatient specialist clinic CDC-94 criteria †	Multidisciplinary rehabilitation with CBT, supervised GET and conventional drugs vs. GET counselling and conventional drugs	3 months therapy assessed 1 year after end of the programme	Rehabilitation, n=60, 53 women, mean age 43 yrs and illness duration 32 months Controls, n=60, 48 women mean age 44 years, illness duration 33 months	Medical Outcomes Study-Short Form-36	No group differences in quality of health, worse bodily pain scores in rehabilitation group than control group
Vos-Vromans et al., 2016 Netherlands	To evaluate the difference in treatment effect between CBT and multidisciplinary rehabilitation (MR)	Rehabilitation centres CDC-94 criteria †	CBT alone (tailored to active or passive patients) vs. Rehabilitation (including CBT and a mix of strategies)	26 and 52 weeks after treatment initiation	CBT, n=60, 47 women, mean age 41 yrs and illness duration mostly ≥ 5 yrs MR, n=62, 50 women, mean age 40, illness duration mostly ≥5 yrs	Checklist Individual Strength fatigue severity subscale	Fatigue improved more in MR than in CBT group After one year the improvement in fatigue score was sustained in MR group, but not in the CBT group.
**Activity pacing:**							
Kos et al., 2015 Belgium	To evaluate effectiveness of activity pacing self-management	Outpatient rehabilitation clinic CDC-94 criteria †	Three individual sessions with either activity-pacing self-management (APS) or relaxation therapy (RT)	At end of therapy at 3 weeks	APS, n= 16 women, mean age 39 yrs RT, n= 17 women, mean age 41 yrs	Canadian Occupational Performance Measure (COPM)	COPM performance and COPM satisfaction scores changed significantly in both group with moderate to high effect size in favour of APS group with 33% and 42% in APS group vs. 14% and none in the RT group.
Pinxsterhuis et al., 2017 Norway	To evaluate the effectiveness of a group-based self-management programme	Primary health care setting CDC-94 criteria † CCD criteria ‡	Self-management educational program vs. treatment as usual (controls)	At 6 months and after 1 year	Self-management, n=71, 67 women, mean age 44 yrs, diagnose for 1-21 yrs Controls n=66, 54 women and diagnosis for 0-17 yrs	SF-36 physical functioning subscale	No group difference in physical functioning after 6 or 12 months

† the Centres for Disease Control and Prevention diagnosis criteria

‡ the Canadian Consensus Criteria for diagnosis

**Table III.** An overview of included papers of patients’ treatment experiences.

References	Aim/research question	Recruitment methods	Setting	Diagnostic criteria	Research design and analysis	Intervention	Sample characteristics	Findings expressed in the authors’ themes
Broughton et al., 2017 UK	To explore experiences of patients who were completing a treatment at specialist clinics	Convenience sampling	Specialist clinics	Diagnosed- criteria not reported	Semi-structured, individual interviews Thematic analysis	Comprehensive treatment including CBT and GET	14 women. 2 men Age 24 to 62 yrs Illness duration 1 to 17 yrs	Journey to specialist care, Things that hinder treatment Support systems
Cheshire et al., 2020 UK	What are the differences and similarities in treatment perceptions and experiences of guided graded exercise among those reporting improvements compared with those deteriorated?	Strategic sampling according to responses to questionnaires showing much improvements/much deterioration	Specialist clinic	Diagnosed—criteria not reported	Semi-structured, individual interviews Thematic analysis	Guided graded exercise self-help (GES)	17 women, 3 men Age 21 to 66 yrs	Getting started and false starts Indeterminate phase of GES Competing commitments Interfering symptoms and comorbid conditions Maintaining motivation
Chew-Graham et al., 2011 UK	What factors are important for patients to engage in a new intervention?	Purposive sampling from an RCT trial	Primary health care	Diagnosed by Oxford criteria	Semi-structured individual interviews Inductive thematic analysis based on data from those in trial arm	Nurse-led rehabilitation program	13 women, 6 men Age 23 to 61 yrs Illness duration 9 months to 16 yrs	Feeling accepted by therapist Own acceptance of diagnosis Accept of (treatment) model
Dennison et al., 2010 UK	To examine participants’ views and experiences of taking part in family-focused cognitive behavioural therapy	All participants in an RCT around three years earlier were invited	Outpatient clinic at a hospital	Diagnosed—criteria not reported	Semi-structured individual interviews Inductive thematic analysis	Either cognitive behavioural therapy or psycho-education	16 out of 46 invited were interviewed 10 women, 6 men Age 16 to 24 yrs	Pre-therapy ideas and expectations Therapy experiences Perspectives on effectiveness
McDermott et al., 2011 UK	To explore hopes and expectations of patients newly referred to a ME/CFS centre	All referred to the service last five months were invited	Specialist clinic	Diagnosed—criteria not reported	Semi-structured individual interviews Constant comparative analysis	Self-help advice to improve symptoms informed by Lifestyle Management Group Program	20 out of 56 invited were interviewed 17 women, 3 men Age 22 to 60 yrs	Needing a diagnosis In search of guidance and support Engaging with complexity to understand the illness In search of hope for the future
Picariello et al., 2017 UK	To explore the reasons for why some patients engage in treatment more than others, and to assess whether CBT meets patients’ needs	Consecutively recruited if finished or in follow-up after treatment	Specialist clinic	Diagnosed—criteria not reported	Semi-structured individual interviews Inductive thematic analysis	Cognitive behavioural therapy (CBT)	13 out of 32 invited were interviewed 11 women, 2 men Age 18 to 64 yrs	Hopes and expectations Real, not imagined Collaborative therapeutic alliance Motivation and engagement Gain and loss Change
Pinxsterhuis et al., 2015 Norway	To elicit participants’ experiences with a patient education programme and its usefulness	All participants in a patient education, self-management programme were invited	Outpatient clinic at a hospital	Centres for Disease Control and Prevention—1994 criteria and Canadian Consensus Criteria	Focus group interviews immediately and 9 months after the program Thematic analysis	A self-management programme based on envelope and self-efficacy theories	10 out of 33 invited were interviewed 8 women, 2 men Age 32 to 57 yrs Illness duration 2.5 to 20 yrs	Experiences of chaos and insecurity Experiences of understanding, acceptance and coping Mediating factors
Reme et al., 2013 US/UK	To explore the experienes of young people after having undergone Lightening process	Recruited through a website for young people with ME	Members of website forum	Oxford criteria	Semi-structured individual interviews Inductive thematic analysis	Lightening Process	8 females, 1 male Age 14 to 26 yrs Illness duration 2 to 12 yrs	Pretreatment thoughts and expectations Experiences with treatment Perspectives on effectiveness

## Results

### First aim: effects of non-pharmacological treatments (NPTs)

#### Study selection

**Figure 1.**
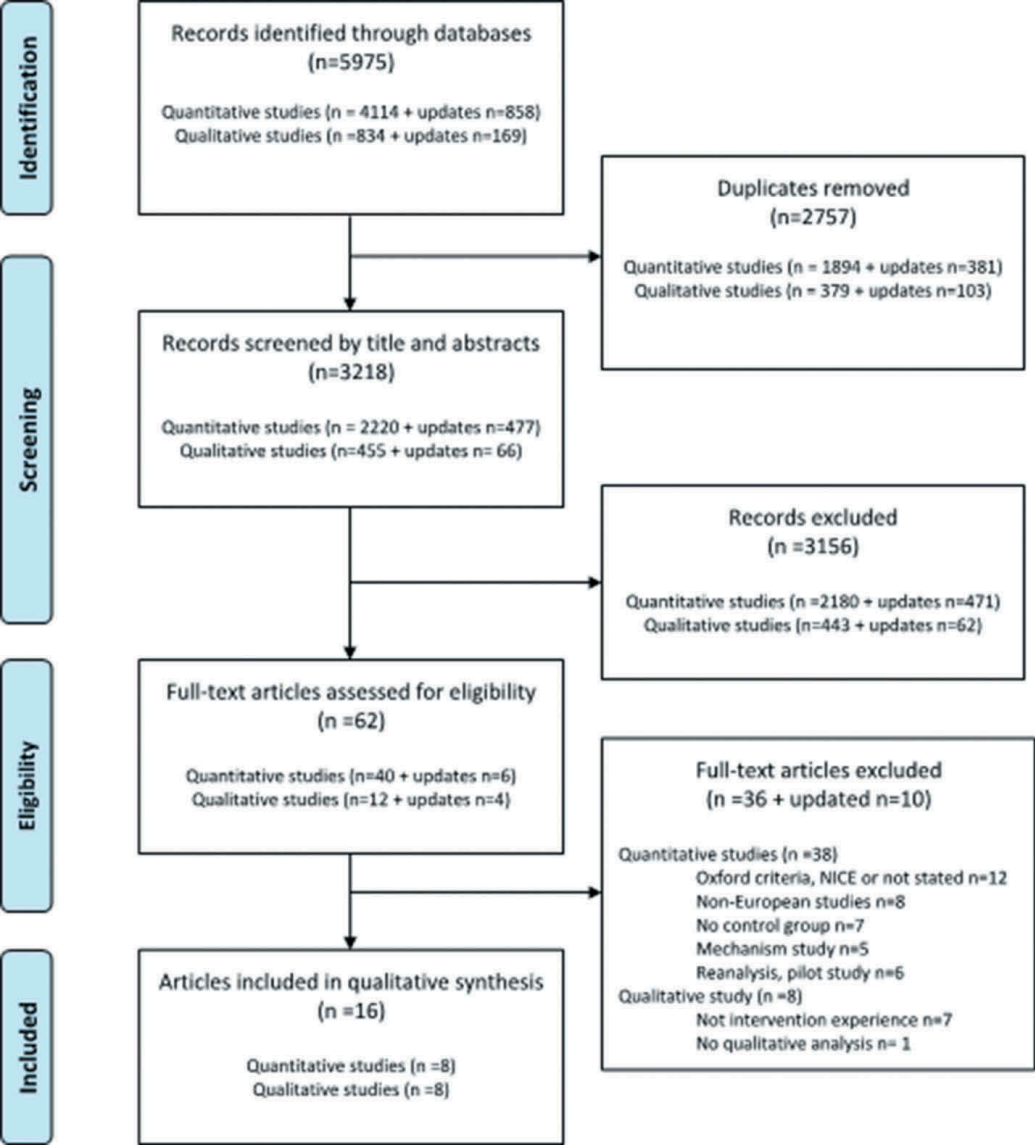
PRISMA flow chart: the selection of scientific papers for the systematic scoping review.

The search strategies on effect studies retrieved 2697 quantitative studies, but records screened by titles and abstracts showed that 2651 papers did not meet the inclusion criteria. Thematically, the latter papers reported about non-ME/CFS illnesses, effects of pharmacological interventions or traditional Chinese medicine, and studies of disease or treatment mechanisms. Forty-six eligible papers were read in full-text. This reading resulted in further exclusion of papers, for reasons shown in Figure 1.

#### Study characteristics

Eight RCT studies were included in the review (Table II). One study fulfiling the inclusion criteria examined 135 children/adolescents with ME/CFS (Nijhof et al., 2012), while the others included 772 adult patients with ME/CFS. Five studies were from the Netherlands (Janse et al., 2018; Nijhof et al., 2012; Tummers et al., 2010, 2012; Vos-Vromans et al., 2016), and one each from Belgium (Kos et al., 2015), Norway (Pinxsterhuis et al., 2017), and Spain (Nunez et al., 2011). The effects of CBT (n = 4), activity pacing (n = 2), and multidisciplinary rehabilitation, including CBT and GET (n = 2) were examined. In all but one study (Tummers et al., 2010), the sample sizes were determined by statistical power calculations based on estimated given clinical relevant changes in the primary outcome variable. Mostly, effects were assessed immediately after completing NPT, except for one study, which assessed outcomes after ﻿1 year (Nunez et al., 2011). Two studies assessed both immediate and long-term effects, either multiple times during ﻿1 year (Vos-Vromans et al., 2016) or after ﻿1 year (Pinxsterhuis et al., 2017). A registry number of Clinical Trial Gov. was not included in any papers, but one study was registered in a Dutch trial registry (Janse et al., 2018).

#### Outcome measures applied in the studies

The primary outcome variables from which power calculations were estimated included; fatigue, physical functioning, dimensions of health-related quality of life and school attendance. The Checklist Individual Strength (CIS) fatigue severity subscale assessed fatigue (Janse et al., 2018; Tummers et al., 2010, 2012; Vos-Vromans et al., 2016). Physical functioning was assessed by the Canadian Occupational Performance Measure (Kos et al., 2015) or the Medical Outcomes Study-Short Form-36 (SF-36) physical functioning subscale (Pinxsterhuis et al., 2017; Tummers et al., 2010). The SF-36 assessed various dimensions of health-related quality of life (Nunez et al., 2011; Vos-Vromans et al., 2016), and school attendance was assessed as a proportion of the normal school-time schedule (Nijhof et al., 2012).

#### Patient characteristics

Seven studies included 772 adult patients with ME/CFS, while the one involving children/adolescents had 135 participants. One study of adults did not report sex for a control group, but among the others, there were 583 women and 111 men. The children/adolescent study included 111 girls and 24 boys (Nijhof et al., 2012). The study participants were recruited from tertiary care (Janse et al., 2018; Kos et al., 2015; Nijhof et al., 2012; Pinxsterhuis et al., 2017; Tummers et al., 2010), primary health care (﻿Nunez et al., 2011; Tummers et al., 2012) or from multiple sources (Pinxsterhuis et al., 2017). The participants’ mean ages varied from 36 to 52 years in the adult samples, and the children/adolescents were aged from 12 to 18 years. Illness duration was not always reported but varied considerably within studies from months to several years, and in one study, time since diagnosis was reported to vary from 1 year to 21 years (Pinxsterhuis et al., 2017).

#### Cognitive behavioural therapy compared with waiting list and usual care controls

Three RCTs, all from the Netherlands, addressed self-instructed CBT programmes either delivered by internet or by self-instruction booklet sent by mail for adult patients. In the study of Janse et al. (2018), no detailed information of the content was provided, but Tummers et al. (2010, 2012) describe the CBT booklet as informing about triggering and maintaining factors, reducing focus on fatigue, establishing sleep routines, managing either an overactive or sedentary physical activity pattern, and the planning of work resumption. Janse et al. (2018) provided the CBT booklet over the internet to a group, with or without guidance from a psychologist, and the groups were compared with waiting list controls. After the intervention period, there was no difference in CIS fatigue severity subscale score between the two CBT groups, but they both improved compared with waiting list controls, and 43% of those receiving CBT achieved normal fatigue scores versus 15% of waiting list controls. In two studies, Tummers and co-workers examined the effects of a self-instruction CBT booklet sent by mail followed by email and face to face consultations with a psychologist in tertiary care (Tummers et al., 2010), and later the same treatment procedure was tried out with mental nurse consultations in a community mental health setting (Tummers et al., 2012). In these studies, no differences in CIS fatigue severity subscale scores were found compared to the control groups of usual care or waiting list controls (participants in both control groups could also receive CBT), but more patients reached clinically relevant changes in fatigue score in the experimental group than those receiving usual care (Tummers et al., 2010) or waiting list controls (Tummers et al., 2012).

Also, a CBT programme provided to children/adolescents and their parents was examined. CBT was delivered as 21 modules on the internet to patients and their parents separately. Trained psychologists provided support by regular email contacts with each individual every other week (Nijhof et al., 2012). The results were compared with usual care, including either group-based multimodal rehabilitation, CBT, GET or a combination of CBT and GET. Both the internet CBT and usual care were delivered over a period of 6 months, and after 6 months 84% improvement in school attendance occurred in the CBT group, versus 52% in the usual care control group.

#### Rehabilitation including cognitive behavioural therapy and graded exercise therapy

Rehabilitation comprised several modalities, among them CBT and GET. In a Dutch study, a multidisciplinary rehabilitation programme (gradual activity reactivation, body awareness therapy, sleep routines following principles of CBT and mindfulness) was compared with a CBT alone programme, with a focus on perpetuating cognitions and behaviour (Vos-Vromans et al., 2016). The rehabilitation programme was provided in the form of weekly individual visits to a physiotherapist and an occupational therapist, and biweekly visits to a psychologist and social worker over a period of 10 weeks, in total 33 h for each individual. In the control group, CBT was provided in 16 sessions of 45–60 min over a period of 6 months. Compared to the rehabilitation programme, CBT was less effective in reducing the CIS fatigue severity subscale score after 4, 14 and 52 weeks. A Spanish study combined conventional symptom-relieving pharmacological therapy with CBT (90 min sessions twice a week for 2.5 months including education about the multifactorial character of ME/CFS, progressive relaxation technique, sleep hygiene, modifying non-adaptive and catastrophic thought patterns) and supervised GET (1 h exercise thrice a week for 3 months, starting with 10 min exercise and adding 5 min for each session until reaching 40 min) compared with conventional drug therapy in combination with counselling about GET (advice to exercise 10 min aerobically and stretching exercises three times a day) (Nunez et al., 2011). Pain reduction assessed by the SF-36 pain subscale was demonstrated in the control group with conventional drug therapy and counselling about GET, whereas the rehabilitation group showed worse SF-36 pain scores after ﻿1 year.

#### Activity-pacing alone or integrated in self-management education

A Belgian study compared the effects of activity-pacing with relaxation therapy (Kos et al., 2015). Three individual therapy sessions of 60 to 90 min a week for three consecutive weeks were provided. Activity pacing, but not relaxation therapy, brought about improvements in self-determined daily activities assessed by the patient-specific assessment method the Canadian Occupational Performance Measure. A Norwegian study based on envelop and self-efficacy theories examined effects of a group-based self-management education programme (goal-setting, modelling self-management skills, guided mastery practice, relaxation exercise). The intervention was delivered in eight sessions of 2.5 h’ duration every other week. The self-management programme did not improve SF-36 physical functioning scores compared with usual care controls either at 6 months or ﻿1 year (Pinxsterhuis et al., 2017).

### Second aim: patients’ treatment experiences

#### Study selection

The search strategies about patients’ treatment experiences retrieved 521 studies, but 505 were excluded, mostly because they did not apply qualitative interviews or described patients’ experiences unrelated to NPTs. After reading 16 papers in full text, eight papers were excluded because they did not refer to treatment experiences or lacked qualitative data analysis (Figure 1). Thus, eight papers were included in the analysis.

Seven studies concerned UK patients (Broughton et al., 2017; Cheshire et al., 2020; Chew-Graham et al., 2011; Dennison et al., 2010; McDermott et al., 2011; Picariello et al., 2017; Reme et al., 2013) and one Norwegians (Pinxsterhuis et al., 2015). The studies explored experiences of psychological treatment programmes as CBT (Dennison et al., 2010; Picariello et al., 2017) or the Lightning Process programme (Reme et al., 2013). Other studies related treatment experiences to education in self-management based on envelop and self-efficacy theories (Chew-Graham et al., 2011; Pinxsterhuis et al., 2015), self-help GET (Cheshire et al., 2020) and multimodal specialist care (Broughton et al., 2017; McDermott et al., 2011).

#### Patient characteristics

Interviews were performed with 162 patients (133 females and 29 males) varying in age from 14 to 66 years, and four studies reported that the participants had been ill from 9 months to 20 years. One study recruited participants from websites for young people with ME/CFS (Reme et al., 2013), one study from primary health care (Chew-Graham et al., 2011), and the other six studies recruited from specialist clinics or outpatient clinics at hospitals.

#### Recognition and support a crucial part of the NPTs

Patients mentioned protracted, distressing history of medical consultations and numerous tests, before being diagnosed as ME/CFS (Broughton et al., 2017; McDermott et al., 2011; Picariello et al., 2017). They had expected that diagnosis would provide authorization for guilt-free downregulation of daily life (Chew-Graham et al., 2011), which was an important step of regaining self-respect and control (Dennison et al., 2010; McDermott et al., 2011). However, it was unclear for them what the diagnosis meant, compounded by lack of knowledge of treatment options and prognosis (McDermott et al., 2011). In the beginning, patients hoped to recover fully and return to how life used to be (Broughton et al., 2017). But over time, few patients expected a miraculous cure, and they rather expressed hopes for more modest symptom relief (Dennison et al., 2010; McDermott et al., 2011), recognition and legitimacy (McDermott et al., 2011). In any case, diagnosis was essential for NPT referral (Chew-Graham et al., 2011), where patients hoped for appropriate advice about how to do the right things to live a life without aggravating symptoms, and thereby balance illness and practical pressures in work and family contexts (Dennison et al., 2010).

Several patients had received advice and treatments that had made them worse (Pinxsterhuis et al., 2015; Reme et al., 2013). Prior negative experiences made patients sceptical about other treatments (Dennison et al., 2010; McDermott et al., 2011; Picariello et al., 2017; Reme et al., 2013), but they were willing to be open and take whatever it cost. This was better than doing nothing, even if health professionals’ advice seemed counterintuitive and caused them to end up in bed for days (Broughton et al., 2017). Often referral to a specialist centre was a last resort (Broughton et al., 2017; Dennison et al., 2010), even sometimes experienced as healing in itself (Picariello et al., 2017). At specialist centres, the validity of ME/CFS was not questioned, and there was acceptance that symptoms were real and serious (Broughton et al., 2017). Health professionals who acknowledged their illness were highly valued by the patients (Broughton et al., 2017; Chew-Graham et al., 2011; Picariello et al., 2017; Pinxsterhuis et al., 2015), who then felt safe (Pinxsterhuis et al., 2015). A key feature was that the staff listened and understood (Chew-Graham et al., 2011; Dennison et al., 2010). Patients developed alliances with these therapists, who they found knowledgeable, inspiring, friendly, supportive and helpful (Picariello et al., 2017). Their services were called a “security blanket” or “lifeboat” (Broughton et al., 2017), a place where patients could talk openly without being judged.

Initially, patients could express a preference for face-to-face consultations with health professionals (Broughton et al., 2017; Picariello et al., 2017), but often NPTs were delivered in group formats. Despite patients’ doubts about group sessions, they often turned out better than expected. Patients found it supportive to share experiences with others, affirming their challenges, and enabling learning from others about illness management (Broughton et al., 2017; Pinxsterhuis et al., 2015; Reme et al., 2013). This support system could collapse after discharge, though, leaving patients uncertain about ability to manage alone (Broughton et al., 2017; Picariello et al., 2017; Pinxsterhuis et al., 2015). They were losing their “safety net”, and would have felt better if offered review appointments after discharge as a “backup” if needed (Broughton et al., 2017; Picariello et al., 2017).

#### Importance of overcoming own scepticism to engage in non-pharmacological therapies

If symptoms were not explained in physical terms, patients concluded that health professionals considered symptoms to be psychological (Chew-Graham et al., 2011). They disliked it when psychological aspects were presented as facts (Chew-Graham et al., 2011; McDermott et al., 2011), and their engagement and compliance diminished (Chew-Graham et al., 2011). In particular, those reporting lack of improvement and attributing their ME/CFS exclusively to organic causes refused psychological explanations (Picariello et al., 2017). This hindered their attempts to balance life in relation to illness and meant that they were told to push themselves more than their body tolerated (McDermott et al., 2011). By contrast, if health professionals presented physiological explanations that matched patients’ own illness models, it helped them to formulate their understandings more clearly, and to form alliances with health professionals (Chew-Graham et al., 2011; Picariello et al., 2017).

Grief over loss of prior life and disruption of plans for future was expressed (Broughton et al., 2017). The need to accept the here-and-now, and willingness to say good-bye to one’s former life and to make changes, were important in engaging fully in treatment (Broughton et al., 2017). Patients could be uncertain that ME/CFS was a correct diagnosis, but participation in NPTs increased their confidence (Pinxsterhuis et al., 2015). Explanation and understanding of symptoms was fundamental to illness acceptance and beginning recovery work (Chew-Graham et al., 2011; Pinxsterhuis et al., 2015). Patients needed to accept that treatment was not curative, that improved coping would help (Pinxsterhuis et al., 2015), and that life henceforth should be lived in a “slow lane” (Broughton et al., 2017). NPTs could make patients more realistic, abandon the search for miracle cures (Pinxsterhuis et al., 2015), and help them to focus on day-to-day goals for their lives (Broughton et al., 2017).

#### Ambiguous experiences of non-pharmacological therapies

It appeared important for patients to build awareness of their condition (Picariello et al., 2017), by learning to plan and manage activity to avoid fatigue triggers and illness fluctuations (Picariello et al., 2017; Pinxsterhuis et al., 2015). Balancing ME/CFS and daily life required adjustment and changes to daily routines (Pinxsterhuis et al., 2015). Patients needed help to achieve this (McDermott et al., 2011). Help in finding the right activity level and implement sleep routines was an important early step (Cheshire et al., 2020; Dennison et al., 2010). Learning pacing, energy conservation, relaxation exercises and coping with negative feelings helped to balance everyday life (Pinxsterhuis et al., 2015).

Acceptance of the psychological explanations on which CBT is founded (Chew-Graham et al., 2011; Pinxsterhuis et al., 2015), was an essential prerequisite to investing effort in CBT (Picariello et al., 2017). The Lightning Process programme also applies mental techniques, but the biological model explaining how the body is affected by thoughts and emotions was mostly accepted (Reme et al., 2013). Although both CBT and the Lightning Process focus on changing patterns of thought and behaviour, for those experiencing both it was confusing that, whereas CBT focuses on accepting illness, coping with its restrictions and adjusting to the new situation, the Lightning Process emphasizes the importance of refusing to allow the illness to take control (Reme et al., 2013). CBT provided a better understanding of the activity patterns that trigger fatigue, and provided skills to manage and plan ahead to prevent overdoing things (Dennison et al., 2010; Picariello et al., 2017), but developing a more consistent daily routine could mean abandoning favoured activities (Picariello et al., 2017). Nevertheless, CBT was considered incomplete, and not addressing all aspects of illness (Dennison et al., 2010). Patients’ experiences suggested the Lightning Process programme was helpful, but they felt it was not specific for ME/CFS and could benefit anyone. Negatively, there was a normative pressure to be positive and not express scepticism embedded in the Lightning Process programme, as the therapists claimed a 100% recovery rate, so patients who did not improve felt blamed for failing to recover (Reme et al., 2013). By practicing energy conservation and relaxation techniques patients felt more in control, but they did not experience better health (Pinxsterhuis et al., 2015). All patients receiving GET found exercising hard work, without immediate benefits. They often felt too unwell to start exercising, and in particular, in the early phases, setbacks were experienced that restricted motivation to continue (Cheshire et al., 2020).

Usually, improvements due to NPTs were small and hardly noticeable (Picariello et al., 2017), and patients felt disheartened by recovering with “baby steps” (Broughton et al., 2017). Nevertheless, patients noted that because of their new skills, the end of the treatment marked a new beginning (Dennison et al., 2010). They felt more in control (Picariello et al., 2017) and able to balance activities and avoid exacerbations (Pinxsterhuis et al., 2015), but had to persevere as progress was slow, particularly for GET. Thus, even starting with light, regular activities, some felt worse with GET, and it could take weeks or months to tolerate even a 10-min walk (Cheshire et al., 2020). In contrast, for some the Lightning Process led to immediate changes, including absence of symptoms and ability to resume former activities (Reme et al., 2013). When participants in NPTs described their expectations for the future, tension between hope and fear was evident (McDermott et al., 2011). They hoped to recover fully (McDermott et al., 2011; Pinxsterhuis et al., 2015) but also feared deterioration (Dennison et al., 2010; McDermott et al., 2011) or recurrence (Picariello et al., 2017).

## Discussion

CBT caused reduced fatigue among adults and led to higher school attendance among adolescents. The results of activity pacing and multimodal rehabilitation programmes, including CBT and GET, were inconclusive. Patients highlighted the importance of recognition and support by health professionals, of making sense of symptoms, and of accepting the situation, enduring slow improvement, and finding the right activity level for everyday life. Nevertheless, patients could find NPTs ambiguous and incomplete.

### A need for more systematic development and testing of non-pharmacological therapies

Many effect studies were excluded because they used diagnostic criteria found inappropriate by EUROMENE, for example, the Oxford criteria (Sharpe et al., 1991), and in particular, this excluded studies examining the effects of GET. In a review of papers until 2004, Friedberg et al. (2007) showed a trend that the effect studies were mostly conducted in the US and England, and then to less extent in the Netherlands and Australia. This trend seems to continue; we excluded many studies from England (not fulfiling the diagnostic criteria set for the present study), the US and Australia. However, we also excluded studies from Asia mainly addressing effects of acupuncture, herbal treatment and meditative exercises. Also prior reviews included mostly European studies, and our reported effects are consistent with those reported in prior reviews across the world and including papers irrespective of case definition. As in our study, previous systematic reviews concluded that CBT reduces fatigue (Chambers et al., 2006; Malouff et al., 2008; Price et al., 2008). Our study suggests that the results may be even better, as the control groups also often received CBT. Unfortunately, the present effect studies of CBT do not include long-time follow-up assessments, so whether the effects are sustained should be addressed. The multimodal rehabilitation programmes included both CBT and GET (Nunez et al., 2011; Vos-Vromans et al., 2016). These rehabilitation programmes vary with respect to content, duration, control groups, and findings. Therefore, like the authors of a recent systematic review who included two studies of rehabilitation in addition to ours (Galeoto et al., 2018), we cannot draw conclusions about effects of rehabilitation. Vos-Vromans’s study (Vos-Vromans et al., 2016) compared rehabilitation (including CBT) with CBT alone, but CBT differed in the two groups. Similarly, Nunez et al. (2011) compared rehabilitation (including supervised GET) with GET counselling without any control of compliance. Therefore, it is also impossible to draw any conclusions about how the CBT and GET components contributed to the effects of the rehabilitation programmes. In fact, a recent meta-analysis concludes that GET has minor positive effects (Larun et al., 2019). Nunez et al. (2011) found a positive effect on pain in counselled GET and worse pain in supervised GET. It seems plausible that those following supervised GET performed more exercise than those getting counselling in GET. This indicates that more exercise can worsen pain. Activity pacing was found efficient in improving physical functioning in one study (Kos et al., 2015), but such improvement was not found after a self-management education programme following principles of activity pacing (Pinxsterhuis et al., 2017). To our knowledge, there has not been any previous published systematic review on the effects of activity pacing for patients with ME/CFS. Taken together, the effects of NPTs, except for CBT, are inconclusive. We carefully compared our findings with other systematic reviews applying broader diagnostic criteria for inclusion of patients with ME/CFS (Chambers et al., 2006; Drachler et al., 2009; Galeoto et al., 2018; Kim et al., 2020; Larun et al., 2019; Malouff et al., 2008; Parslow et al., 2017; Price et al., 2008). The conclusions of these systematic reviews are in line with ours, so our assumption that diagnosis criteria could explain the positive effects reported in some prior studies, for example, from CBT, is not supported.

Reviewing patients’ treatment experiences, patients found acknowledgement, reassurance and support by health professionals to be important, and they felt safer if they had a follow-up after completing NPTs. Previous reviews of adolescent (Parslow et al., 2017) and adult studies (Drachler et al., 2009) have, consistent with our findings, reported that patients are frustrated about the disputes over the nature of ME/CFS, find recognition by health professionals valuable, resist psychological explanations, and strive to find effective self-management strategies. The interviews highlight that health professionals meet the patients’ needs for recognition, but they also have negative experiences. Health professionals are “instruments” in delivering NPTs, and what is delivered appears inseparable from how it is delivered and by whom. Therefore, attitudes and behaviours of health professionals matter. It may be very challenging to meet patients when they have had negative experiences of former consultations with health professionals, as demonstrated by accounts of patients with chronic musculoskeletal pain (Mengshoel et al., 2019). In the words of Hurwitz et al (2004, p. 90), “personal healing does not merely depend on what happens during therapy, but how therapy becomes an episode in a larger narrative of illness and recovery experiences.” In addition, patients conclude that NPTs are incomplete. They tend to feel uncertain about the meaning of illness and express a need to find effective self-management strategies, and this has to be given priority when designing new NPTs. It seems that patients search to understand how to connect themselves and their lives to a non-comprehensible illness and its fluctuating course. Therefore, more knowledge about these issues has to be pursued by future research.

### Methodological issues

The content and delivery of NPTs varied considerably in the studies, and any theoretical reasons for including the various components are not given. As NPTs are based on various theoretical assumptions, each NPT should first be tested to see if it is successful in achieving what it theoretically is supposed to do, and whether the changes are relevant in helping patients with ME/CFS. If successful in both respects, other components could be added step by step and tested in accordance with the framework of developing complex interventions (Craig et al., 2013; Richards & Hallberg, 2015). Future RCTs should also include long-time follow-up and cost-benefit analysis, and in particular, more studies on the role of NPTs for children and adolescents are needed.

The studies of CBT showed rather consistent results in having an effect on modifying fatigue, while the effects of activity pacing and multimodal rehabilitation were inconsistent. However, a timely question is whether the outcome measures were appropriate in evaluating whether the intentions of the NPTs, and what was found relevant for patients, were reached. At present, generic instruments, such as SF-36 and the CIS fatigue subscale, mostly assessed effects. A systematic literature review by Haywood et al. (2012) examined whether patient-reported measures, including those used in our review, had undergone rigorous, scientific evaluations in patients with ME/CFS. No evidence was found that patients had been involved in evaluating the relevance of any questionnaire, the content validities of which are therefore questionable. Likewise, measures showed little or moderate responsiveness to NPTs, which is essential in detecting effects. Another issue to be considered is how to interpret statistically significant differences in relation to clinical importance. It is questionable how much change is needed in an instrument’s scale to make it both clinically relevant and meaningful for patients (Angst et al., 2017). For future research, we recommend developing robust patient-reported disease-specific measures in collaboration with patients, and international consensus should be reached about the use of such instruments in making findings comparable across studies.

Patients with ME/CFS are not homogenous. Severely affected patients are house- or bedbound, while others, well represented in the effect studies we identified, struggle to maintain their living patterns. We did not find any studies on NPTs for severely affected patients. One reason for this may be that RCT designs are not appropriate in evaluating effects of such therapies for severely affected patients. To attempt to close this knowledge gap, case studies are high priority. Interviews with severely afflicted patients about their illness experiences, and interviews with patients that have become better after being bed- or house bond about what mattered for their progress, are needed to develop meaningful NPTs in the future. An alternative means of testing such new NPTs could be to apply single subject experimental designs in which a subject serves as his/her own control (Bates, 1996; Zhan & Ottenbacher, 2001). Patient-specific measures, such as Canadian Occupational Performance Measure as used by Kos et al. (Kos et al., 2015) can be appropriate to examine eventual progress but are less easy to use because of the semi-structured format. In terms of clinical practice, the semi-structured format has the advantage of being already a first step in the communication with the patients, but in research, reliability can be an issue when the assessors are not well trained.

### Methodological considerations of the scoping review

A limitation of our study is that we excluded grey papers. By excluding doctorial theses, we noticed that we lost monographs of qualitative studies. However, the limited time for conducting the review made it impossible to analyse data from such theses. A strength of our scoping review is that the authors have various backgrounds, come from various European countries, and are active researchers within this field. Our strategies follow a well-defined structure, and hopefully, our findings are presented in a transparent way, so the trustworthiness should be appropriate. The majority of the included studies were from North-Western Europe. The strict inclusion criteria might have excluded papers from other European regions. However, with respect to the excluded effect studies, they were mostly conducted in the UK. The qualitative studies were not selected on the basis of diagnostic criteria, and all except one were UK studies, and thus all were conducted in the North-Western Europe. We may have overlooked some studies, but also prior systematic reviews include papers mostly from North-Western Europe, and studies from Eastern Europe and the South would be welcomed.

In conclusion; this scoping review underlines the importance of more positive social attitudes towards ME/CFS in general, and by health professionals in particular. But the patients express ambiguity towards the contents of the programmes and consider them to be incomplete. CBT was found to relieve fatigue, but its long-term effects need more investigation. Studies on activity-pacing and rehabilitation are scarce, and the effects are inconsistent. Patients, clinicians and researchers should collaborate in critically scrutinizing existing patient reported outcome measures, and in systematically developing disease-specific measures and NPTs that are tailored to the particular needs of patients with ME/CFS.
